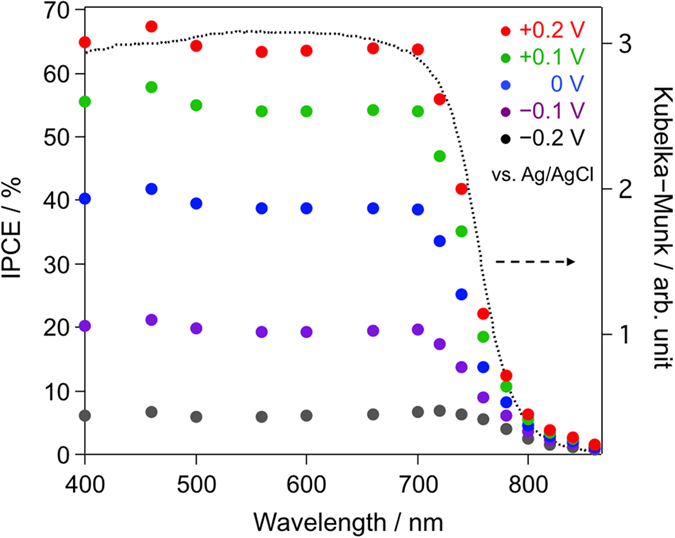# Low-Temperature Synthesis of Bismuth Chalcohalides: Candidate Photovoltaic Materials with Easily, Continuously Controllable Band gap

**DOI:** 10.1038/srep32664

**Published:** 2016-09-07

**Authors:** Hironobu Kunioku, Masanobu Higashi, Ryu Abe

**Affiliations:** 1 Department of Energy and Hydrocarbon Chemistry, Graduate School of Engineering, Kyoto University, Katsura, Nishikyo-ku, Kyoto 615-8510, Japan; 2 CREST, Japan Science and Technology Agency (JST), Kawaguchi, Saitama 332-0012, Japan

## Abstract

Although bismuth chalcohalides, such as BiSI and BiSeI, have been recently attracting considerable attention as photovoltaic materials, the methods available to synthesize them are quite limited thus far. In this study, a novel, facile method to synthesize these chalcohalides, including BiSBr_1−*x*_I_*x*_ solid solutions, at low temperatures was developed *via* the substitution of anions from O^2−^ to S^2−^ (or Se^2−^) using bismuth oxyhalide precursors. Complete phase transition was readily observed upon treatment of BiOI particles with H_2_S or H_2_Se at surprisingly low temperatures of less than 150 °C and short reaction times of less than 1 h, producing BiSI and BiSeI particles, respectively. This method was also applied for synthesizing BiSBr_1−*x*_I_*x*_, where continuous changes in their band gaps were observed depending on the ratio between iodine and bromine. The composition of all elements (except oxygen) in the chalcohalides thus produced was almost identical to that of the oxyhalide precursors, attributed to the suppressed volatilization of halogens at such low temperatures. All chalcohalides loaded on FTO clearly exhibited an anodic photocurrent in an acetonitrile solution containing I^−^, attributed to their n-type nature, e.g., the BiSI electrode exhibited high IPCE (64% at 700 nm, +0.2 V vs. Ag/AgCl).

Bismuth-based non-oxide compounds, such as halides (e.g., BiI_3_^1^, A_3_Bi_2_I_9_ (where A = Cs or CH_3_NH_3_)^2^), chalcogenides (e.g., Bi_2_S_3_^3^, Bi_2_Se_3_^4^), and chalcohalides (e.g., BiSI^5^, [Bi_2_Te_2_Br](AlCl_4_)^6^), exhibit fascinating electrical, magnetic, and optical properties. Especially, bismuth-based chalcohalides like BiSI or BiSeI have attracted significant attention of late as photovoltaic (PV) materials^7^,^8^,^9^, attributed to their excellent semiconducting nature and narrow band gap (BiSI: 1.59 eV^10^ and BiSeI: 1.29 eV^11^), which enable the utilization of a wide range of solar spectrum. In addition, fine, tailored tuning of bands is strongly expected to be possible *via* arbitrary substitution between iodide and bromide, examples of which have been reported for some oxyhalide systems such as BiOBr_1−*x*_I_*x*_^12^. Furthermore, the non-toxic, earth-abundant nature of Bi make such bismuth-based chalcohalides promising alternatives to hybrid perovskites (e.g., CH_3_NH_3_PbI_3_^13^,^14^) or chalcopyrites (e.g., Cu(In, Ga)Se_2_^15^) PVs, both of which have been extensively investigated.

However, there are only a few reports on the synthesis of BiSI and BiSeI. For example, the Bridgman–Stockbarger technique^16^,^17^ and vapor-phase growth^18^,^19^ have been employed for producing single-crystal BiSI and BiSeI. However, both these synthetic techniques require high temperatures (~400 °C) and complicated equipment. Solvothermal methods that produce BiSI particles at 160–180 °C have been demonstrated. Zhu *et al*. have prepared BiSI particles by employing a solvothermal method at 180 °C for 1 day; however, the obtained sample included obvious impurity phases^20^. Fa *et al*. have also synthesized BiSI particles *via* a solvothermal method even at 160 °C; however, a long time reaction of 30 h is indispensable for obtaining sufficiently crystalline samples^21^. Thus, solvothermal methods enable low-temperature synthesis of BiSI particles; however, they always require both long reaction times and high-pressure conditions. Recently, Hahn *et al*. have prepared BiSI photoanodes on a conductive glass substrate (FTO) *via* spray pyrolysis and applied them as PV materials combined with an iodine redox or p-type CuSCN^7^,^8^. Although rod-like BiSI particles can be produced at a low temperature of around 250 °C, by this method, the decreased iodine content was observed even at that temperature (e.g., ~0.8 of I/Bi at 250 °C, determined *via* EDX analysis); further decrease in the I/Bi value was observed at higher temperatures. Surprisingly, there is only one study^22^ about the synthesis of bismuth chalcohalide BiSBr_1−*x*_I_*x*_ solid solution, in which BiSBr and BiSI particles were mixed and heated in a sealed tube under vacuum at a relatively higher temperature (~400 °C). However, this method involves multi-step reactions, including synthesis of the precursors (BiSBr and BiSI) and a long preparation time (~1 week). Furthermore, the sealed-tube procedure is indispensable for obtaining products with the intended compositional ratio *via* the suppression of the volatilization of halogens (probably that of bromine with a boiling point lower than that of iodine) at such high temperatures.

As described above, one of the key issues in the synthesis and application of such Bi chalcohalides involves the volatilization of halogens during conventional synthesis, which is associated with heating at a high temperature. Therefore, it is imperative to develop a new, facile method for synthesizing chalcohalides at significantly low temperature for the purpose of further investigation and applications.

In this study, BiSI and BiSeI particles were readily synthesized by simply heating BiOI particles under H_2_S or H_2_Se gas at quite low temperatures (<150 °C) for a short time (<1 h) *via* the substitution of anions from O^2−^ to S^2−^ (or Se^2−^). BiSBr_1−*x*_I_*x*_ solid solution was also synthesized by this low-temperature anion substitution, with a retained compositional ratio between Br and I, attributed to the successful suppressed volatilization of halogens at such low temperatures. The photoanodes of BiSI prepared by this new synthesis method exhibited high incident photon-to-current conversion efficiency (IPCE) values, indicating the possibility of high-performance PVs based on such Bi chalcohalides.

## Results and Discussion

### Synthesis of BiSI from BiOI *via* low-temperature treatment with diluted H_2_S

**Figure 1:**
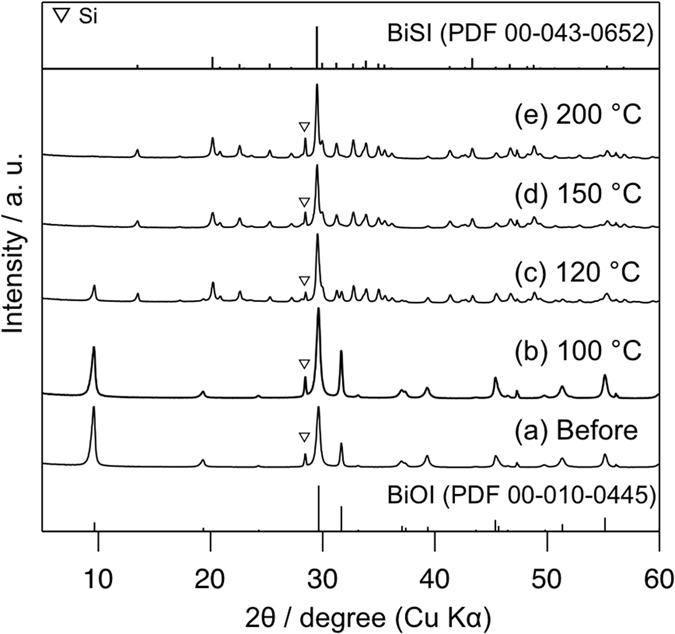
XRD patterns of the prepared samples (**a**) before and after heat treatment with 5% H_2_S gas at (**b**) 100 °C, (**c**) 120 °C, (**d**) 150 °C, and (**e**) 200 °C.

Figure 1 shows the powder XRD patterns of BiOI samples before and after heat treatment with H_2_S/Ar gas at different temperatures (100–200 °C) for 1 h. Surprisingly, several peaks were observed even at a very low temperature of 120 °C, attributed to orthorhombic BiSI (PDF 00-043-0652), and the complete phase transition from BiOI to BiSI was observed at 150 °C. Although no peak attributed to impurities was observed up to 200 °C, high temperature resulted in the formation of impurities poor in iodine, such as Bi_12.7_S_18_I_2_ (e.g., at 300 °C, see Figure S1 in Supporting Information), clearly attributed to the volatilization of iodine. The EDX analysis on the BiSI sample prepared at 150 °C indicated the presence of iodine as almost stoichiometric ratio to Bi as same as to in the BiOI precursor (Table S1), indicating that such a low-temperature reaction does not change the molar ratio of I/Bi even under atmospheric conditions (i.e., without sealed-tube combustion). In addition, the stoichiometric content of sulfur (S) was confirmed in the BiSI sample prepared at 150 °C (see Table S1), indicating the successful substitution between O^2−^ and S^2−^ *via* the current low-temperature treatment. It was also confirmed that this anion substitution was completed within a quite short period. XRD analysis (Figure S2) revealed that the phase transition from BiOI to BiSI was nearly accomplished within 15 min after H_2_S treatment at 150 °C; however, small peaks attributed to BiOI were still observed.

**Figure 2:**
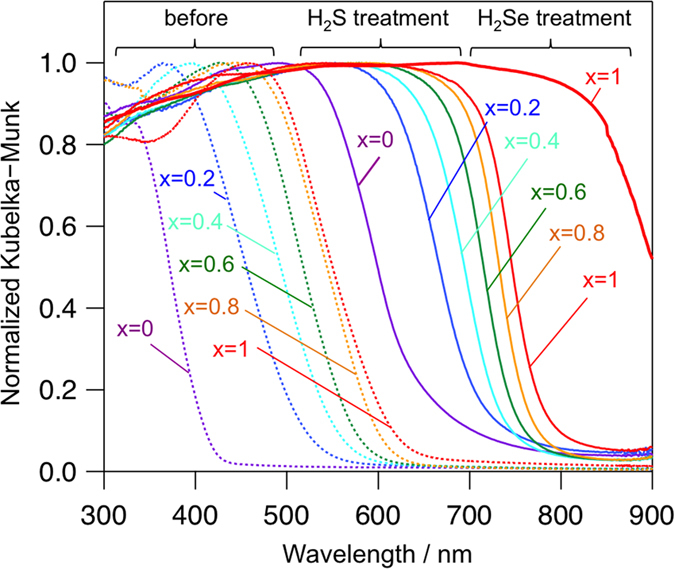


As shown in Fig. 2 (*x* = 1), after H_2_S treatment at 150 °C for 1 h, the UV–vis diffuse reflectance spectra of BiOX clearly changed, in which the absorption edge significantly shifted from ~630 nm to 780 nm, again supporting the successful phase transition from BiOI to BiSI. As shown in the SEM images in Figure S3, after heat treatment, particle morphology significantly changed from plate-like (BiOI) to rod- or block-like (BiSI) shapes, clearly reflecting phase transition.

The above results obtained from the facile synthesis of BiSI motivated us to apply this protocol to the synthesis of bismuth selenide iodide (BiSeI). For reaction with toxic gaseous H_2_Se, a closed system was utilized, in which H_2_Se gas was generated by the reaction between ZnSe and HCl in a vial. As shown in Figure S4, the XRD patterns of BiOI after H_2_Se heat treatment at 150 °C were in good agreement with those of orthorhombic BiSeI (PDF 00-044-0162), indicating successful phase transition *via* H_2_Se treatment, similar to the case of H_2_S treatment. As shown in Fig. 2, the UV–vis diffuse reflectance spectra of the prepared sample after H_2_Se treatment (*x* = 1) exhibited longer absorption (>900 nm) as compared with that of the prepared sample after H_2_S treatment. Previously, Ganose *et al*. have reported that the band gap of BiSeI is smaller than that of BiSI, attributed to the contribution of Se 4p orbitals to the formation of the valence band by DFT calculations^9^. The prepared BiSeI exhibited an absorption edge at ~940 nm (Figure S5), which is slightly shorter than that reported previously^23^, while the absorption spectra exhibited a pronounced background level, attributed to impurities such as Bi_2_Se_3_ or other reduction species. The fine control of reaction time, as well as temperature and gas concentration, would provide high-purity bismuth selenide halides.

To the best of our knowledge, this is the first demonstration of phase transition from oxyhalides to chalcohalides *via* the substitution of anions from O^2−^ to S^2−^ or Se^2−^. It is surprising that this method not only demonstrates successful, facile transition, but also requires low temperature (~150 °C) for transition. As shown in Figure S6, the series of BiOX materials exhibited a Sillén-phase structure, in which two halides were present in relatively open spaces between the cationic [Bi_2_O_2_]^2+^ layers; these layers were electrostatically attracted to each other, affording a two-dimensional-layered structure. Thus, such a low-temperature phase transition is attributed to the facile intercalation of H_2_S molecules into the interlayer spaces, at which H_2_S can effectively react with O^2−^ in the [Bi_2_O_2_]^2+^ layers.

### Synthesis of BiSBr_1−*x*_I_*x*_ solid solution through phase transition from BiOBr_1−*x*_I_*x*_

**Figure 3:**
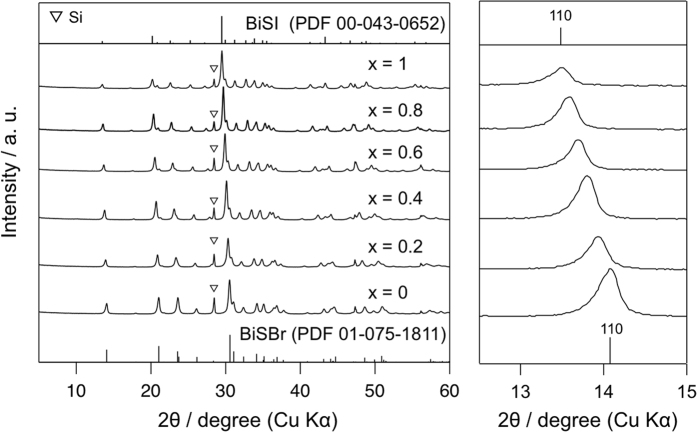


These results motivated us to apply this low-temperature phase transition to the synthesis of BiSBr_1−*x*_I_*x*_ solid solutions from BiOBr_1−*x*_I_*x*_, as the original ratios of iodine to bromine (I/Br), as well as those of bismuth to halogens (Bi/(I+Br)), are expected to be retained after phase transition, attributed to the suppressed volatilization of halogens. Figure 3 shows the XRD patterns of the BiOBr_1−*x*_I_*x*_ samples after H_2_S treatment at 150 °C for 1 h. The diffraction pattern of the product obtained from BiOBr (*x* = 0) showed good agreement with that of orthorhombic BiSBr (PDF 01-965-1811), indicating successful phase transition *via* H_2_S treatment, similar to the case of BiOI to BiSI. The (110) diffraction peaks (shown in the enlarged part in Fig. 3) monotonically shifted toward low angles with increasing *x* value. The lattice constants as calculated by the Le Bail method^24^ increased almost linearly with increasing *x* values (Figure S7), satisfying Vegard’s rule^25^. Moreover, EDX analysis confirmed that the I/(Br+I) values in the products increase with increasing *x* value in the BiOBr_1−*x*_I_*x*_ precursors (see Figure S8). These results clearly indicate the formation of BiSBr_1−*x*_I_*x*_ solid solution *via* anion substitution from BiOBr_1−*x*_I_*x*_, with the retention of the original halogen contents. To the best of our knowledge, there is only one study^22^ reported for the synthesis of a series of BiSBr_1−*x*_I_*x*_ solid solutions. However, the reported method requires multistep sealed-tube combustion, in which both high temperatures (~400 °C) and long reaction times (~1 week) are indispensable. In addition, excess amounts of halogen precursors are used in this method, probably to compensate for the volatilization of halogen during high-temperature calcination. In stark contrast, with the H_2_S heat treatment described herein, a series of BiSBr_1−*x*_I_*x*_ solid solutions were synthesized *via* a one-step reaction at low temperature and in a very short time. As shown in Fig. 2, the absorption edges of BiSBr_1−*x*_I_*x*_ continuously shifted toward long wavelengths with increasing *x* values; their indirect band gaps calculated by the Tauc plot decreased from 1.71 to 1.51 eV (see Figure S9), indicating higher contribution of the I 5p orbitals, as compared with that of Br 4p, to valence band formation. By the comparison of the absorption spectra of BiSBr_1−*x*_I_*x*_ with each corresponding spectrum of BiOBr_1−*x*_I_*x*_, anion substitution (from O^2−^ to S^2−^) resulted in the significant shift of the absorption edges toward long wavelength in the range of 150–260 nm (Fig. 2), suggesting the contribution from high-energy S 3p orbitals to valence band formation. First-principles band calculation indeed confirmed the significant contribution of S 3p orbitals to valence band formation in both cases (Figure S10); the valence band maximum (VBM) of BiSBr was composed of S 3p orbitals mixed with Br 4p, while that of BiSI was predominantly occupied by I 5p. Because the VBM of original BiOBr mainly consisted of Br 4p, albeit clearly mixed with O 2p (Figure S11), the substitution of anions from O^2−^ to S^2−^ undoubtedly affected the VBM significantly, resulting in the significant decrease of band gap (1.1 eV, Figure S9). On the other hand, as the VBM of the original BiOI exclusively consisted of I 5p orbitals, attributed to the significantly higher energy of I 5p orbitals as compared with that of O 2p, the substitution of anions from O^2−^ to S^2−^ in BiOI exerted less impact on the VBM, indeed resulting in a significantly lesser decrease of band gap (0.33 eV). As a result, for solid solutions, the decrease of band gap *via* phase transition became less prominent with increasing *x* values (i.e., increasing contribution of I 5p orbitals) (Figure S9).

### Application to BiSBr_1−x_I_x_ electrode

As discussed above, a novel anion substitution reaction, which can readily produce solid solutions of BiSBr_1−*x*_I_*x*_, was demonstrated under atmospheric conditions at low temperature (~150 °C). As such bismuth chalcohalides, especially BiSI with a band gap of 1.51 eV, have attracted considerable attention as PV materials^9^, thin film photoelectrodes of BiSI and BiSBr_1−*x*_I_*x*_ were fabricated on a conducting substrate, and their performance was evaluated.

**Figure 4:**
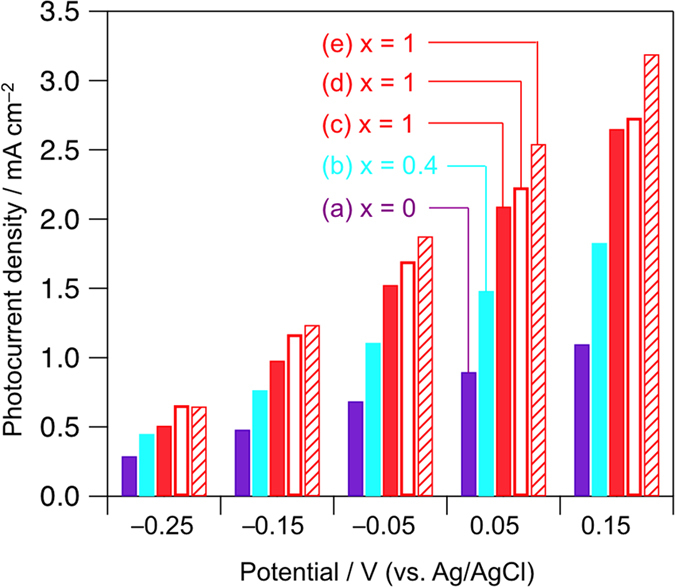
Current–potential relationship for the BiSBr_1−*x*_I_*x*_ electrode in an acetonitrile solution containing 0.1 M NaI under visible-light irradiation. Electrodes (**a–c**) were prepared by coating BiSBr_1−*x*_I_*x*_ particles on FTO. Other electrodes were prepared by the phase transition of BiOBr_1−*x*_I_*x*_ coated on a substrate by (**d**) the squeegee method or (**e**) EPD under H_2_S flow at 150 °C.

Figure 4 shows the current–potential relationship for BiSBr_1−*x*_I_*x*_ (*x* = 0, 0.4, 1) electrodes in an acetonitrile solution containing 0.1 M of NaI under visible-light irradiation. These electrodes were basically fabricated *via* the simple squeegee method, in which an aqueous slurry of BiSBr_1−*x*_I_*x*_ particles was coated on an FTO substrate by using a glass rod. All electrodes clearly exhibited anodic photocurrent, attributed to n-type nature. High *x* values afforded high photocurrent density, undoubtedly attributed to the high number of absorbed photons. Although clear anodic photocurrent was observed *via* the simple squeegee of the aqueous slurry of BiSI particles on FTO, the photocurrent density significantly increased with increased applied potentials (Fig. 4c), implying the existence of considerably high resistance; intrinsic resistance in semiconductor bulk and/or extrinsic resistance existing at grain boundary. Thus, phase transition is attempted to be triggered from BiOI to BiSI directly on FTO for enhancing conductivity among the BiSI particles, as well as between the BiSI particles and the FTO substrate. The BiSI electrode thus prepared by heating the BiOI-coated FTO (*via* simple squeegee of BiOI particles) under H_2_S/Ar at 150 °C for 10 min (Fig. 4d and Figure S12 for the XRD pattern) exhibited an appreciably higher photocurrent density specifically under low applied potentials (from −0.25 to +0.05 V vs. Ag/AgCl). The enhanced photocurrent strongly suggests reduced grain boundary resistivity, attributed to the partial formation of junctions between adjacent particles concurrently with phase transition. Furthermore, electrophoretic deposition (EPD) method^26^ was employed for fabricating a more densely packed BiOI precursor on FTO, which was also subjected to direct phase transition with H_2_S/Ar (Figure S13 for the XRD pattern). The prepared BiSI electrode exhibited the highest photocurrent at all potentials (Fig. 4e), clearly attributed to the reduced resistance and/or the increased number of absorbed photons by densely packed particles. Figure 5 shows the IPCE spectra of the BiSI electrode at various applied potentials as a function of irradiation wavelength. The spectral curves were in agreement with the photoabsorption of the BiSI powder, indicating that the photocurrent is attributed to the band gap transition of BiSI. The obtained IPCE value (e.g., 64% at 700 nm at +0.2 V vs. Ag/AgCl) was significantly higher than that reported previously for BiSI photoelectrodes (e.g., ~38% at 500 nm at +0.4 V vs. Ag/AgCl)^7^ under similar conditions, highlighting the potential utility of the BiSI (or other solid solutions) electrodes prepared herein to highly efficient PV materials in the future. Nevertheless, the current–potential relationship for the BiSI electrodes indicated the presence of high resistivity as discussed. Hence, it is imperative to further examine not only improvement in preparation procedures of electrodes for decreasing extrinsic resistances, but also the control of the intrinsic property of BiSI bulk (e.g., controlling donor density or decreasing crystal defects), for facilitating charge transport for the purpose of developing highly efficient PV cells based on these materials.

## Conclusion

In summary, a new, facile method was developed for synthesizing bismuth chalcohalides like BiSI, BiSeI, and BiSBr_1−*x*_I_*x*_ solid solution *via* low-temperature (<150 °C) phase transition from the corresponding oxyhalides within a short time period (<1 h). This method permitted the synthesis of bismuth chalcohalides without any complicated procedure (e.g., sealed-tube combustion), as well as the continuous tailoring of band gaps by controlling halide composition, i.e., the ratio between Br^−^ and I^−^, both of which significantly contribute to valence band formation. Such precise control of halide composition was accomplished because of the occurrence of phase transition at unbelievably low temperatures and within short time, entirely suppressing the volatilization of the halogen component. As demonstrated, the direct phase transition on the FTO substrate resulted in the performance improvement of BiSI photoanodes. Phase transition on conductive plastic substrates will also open avenues for the low-cost production of flexible, high-performance PV devices, e.g., by employing roll-to-roll manufacturing.

## Methods

### Synthesis of BiOBr_1−*x*_I_*x*_ precursors

Particles of BiOBr_1−*x*_I_*x*_ (*x* = 0, 0.2, 0.4, 0.6, 0.8, 1), serving as precursors to BiSBr_1−*x*_I_*x*_ particles, were synthesized *via* a soft chemical method according to a previous study^27^. First, a mixture of NaBr and NaI with a molar ratio of 1 − *x: x* (3 mmol in total) was dissolved in distilled water (37.5 mL) with CH_3_COONa (6 mmol, 98.5%, Wako). Second, another solution, serving as the bismuth source, was prepared in parallel, in which Bi(NO_3_)_3_·5H_2_O (3 mmol, 99.9%, Wako) was dissolved in glacial acetic acid (2.5 mL, 99.5%, Wako). Next, this solution with the Bi source was added dropwise into the first solution containing halides with vigorous stirring using a magnetic stirrer. The mixture was then continuously stirred for 20 h at room temperature. Finally, the precipitate produced was purified by centrifugation, thoroughly washed two times with distilled water, and finally dried at 80 °C for 5 h in air.

As shown in Figure S14, the XRD patterns of the obtained BiOBr and BiOI samples were in good agreement with those in database (PDF 01-085-0862 and PDF 00-010-0445, respectively). With increasing *x* value in the BiOBr_1−*x*_I_*x*_ samples, the diffraction peaks (e.g. (001) diffraction observed in the enlarged view) shifted toward high angles, and the lattice parameter *a* increased almost linearly with increasing *x* value (Figure S15), indicating the successful formation of solid solutions between BiOBr and BiOI, as has previously been reported^27^. Elemental analysis by EDX confirmed that each sample contains Br and I in a ration close to the intended one of 1 − *x: x*.

### Synthesis of BiSBr_1−*x*_I_*x*_

The obtained BiOBr_1−*x*_I_*x*_ particles (0.5 g) were placed in an alumina boat and heated in a tube furnace under a stream of 5% H_2_S/Ar (60 mL min^−1^) at 100–200 °C for 1 h.

### Synthesis of BiSeI

The BiOBr_1−*x*_I_*x*_ particles thus prepared were also treated by H_2_Se gas. For minimizing the risk of exposure to the more toxic H_2_Se gas, the reaction was conducted in a closed system, in which H_2_Se gas was generated *in situ via* the reaction with ZnSe and HCl in a vial^28^. First, two vials (15 mL)—one containing 0.2 g of BiOI particles and the other containing 0.6 g of ZnSe (Aldrich, 99.99%)—were sealed with silicone septa caps and connected to each other *via* a Teflon-made tube passing through the septa caps. Second, the insides of the vials were purged with Ar gas. Third, the vial containing BiOI particles was heated at 150 °C in an aluminum dry bath. Simultaneously, 4 mL of 8 M HCl was injected into the other vial containing ZnSe particles, followed by heating the vial at 150 °C on a hotplate for producing H_2_Se gas. Finally, after 1 h of heating the sample under the generated H_2_Se gas, these vials were thoroughly purged by Ar for removing the unreacted H_2_Se gas.

### Characterization

The samples thus prepared were characterized by powder X-ray diffraction (XRD; MiniFlex II, Rigaku, Cu Kα), scanning electron microscopy (SEM; NVision 40, Carl Zeiss-SIINT), energy-dispersive X-ray spectroscopy (EDX; X-max, Oxford Instruments), and UV–visible diffuse reflectance spectroscopy (V-650, Jasco). The lattice parameters were determined by Le Bail analysis^24^ with JANA2006^29^ program.

### Preparation of photoelectrodes

The BiSBr_1−*x*_I_*x*_ electrode was prepared by coating particles BiSBr_1−*x*_I_*x*_ on a fluorine-doped tin oxide (FTO) substrate *via* the squeegee method. In addition, the BiSI electrode was prepared by coating the BiOI precursor *via* the squeegee method or electrophoretic deposition (EPD)^26^ on FTO. Then, the electrodes were dried in air at room temperature and were subsequently heated under a stream of 5% H_2_S/Ar (10 mL min^−1^) at 150 °C for 10 min.

### Photoelectrochemical measurements

Photoelectrochemical (PEC) measurements were performed using a three-electrode cell. The prepared electrode, Pt wire, and Ag/AgCl were used as the working, counter, and reference electrodes, respectively. The potential of the working electrode was controlled using a potentiostat (VersaSTAT3, Princeton Applied Research). A current–potential curve was measured in an acetonitrile solution containing 0.1 M NaI under chopped visible light emitted from a 300W-Xe lamp (Cermax) fitted with a cut-off filter.

### Calculation

The calculations were performed with the Cambridge Serial Total Energy Package (CASTEP)^30^. Energy was calculated by the generalized gradient approximation (GGA) of DFT, as proposed by Perdew, Burke, and Ernzerhof (PBE). The electron configurations of the atoms were as follows: Bi, 6s^2^6p^3^; O, 2s^2^2p^4^; S, 3s^2^3p^4^; Br, 4s^2^4p^5^; and I, 5s^2^5p^5^. The electronic states were expanded using a plane-wave basis set with a cutoff energy of 340 eV. The *k*-point set was 4 × 4 × 3. Geometry optimization was performed before calculating the electronic structures using the Broaden–Fletcher–Goldfarb–Shanno (BFGS) algorithm.

## Additional Information

**How to cite this article**: Kunioku, H. *et al*. Low-Temperature Synthesis of Bismuth Chalcohalides: Candidate Photovoltaic Materials with Easily, Continuously Controllable Band gap. *Sci. Rep.* **6**, 32664; doi: 10.1038/srep32664 (2016).

## Supplementary Material

Supplementary Information

## Figures and Tables

**Figure 5:**