# Supplementary material for: Low-Temperature Synthesis of Bismuth Chalcohalides: Candidate Photovoltaic Materials with Easily, Continuously Controllable Band gap
Source: Sci Rep. 2016 Sep 7;6:32664. doi: 10.1038/srep32664 (PMC5013401; doi:10.1038/srep32664)
Supplement: Supplementary Information [file srep32664-s1.pdf]

Supporting information

Low-Temperature Synthesis of Bismuth Chalcohalides: Candidate Photovoltaic Materials with Easily, Continuously Controllable Band gap

Hironobu Kunioku[1], Masanobu Higashi[1], Ryu Abe[1,2,*]

[1]Department of Energy and Hydrocarbon Chemistry, Graduate School of Engineering, Kyoto University

Katsura, Nishikyo-ku, Kyoto 615-8510, Japan

[2]CREST, Japan Science and Technology Agency (JST), Kawaguchi, Saitama 332-0012, Japan

[*]*ryu-abe@scl.kyoto-u.ac.jp*

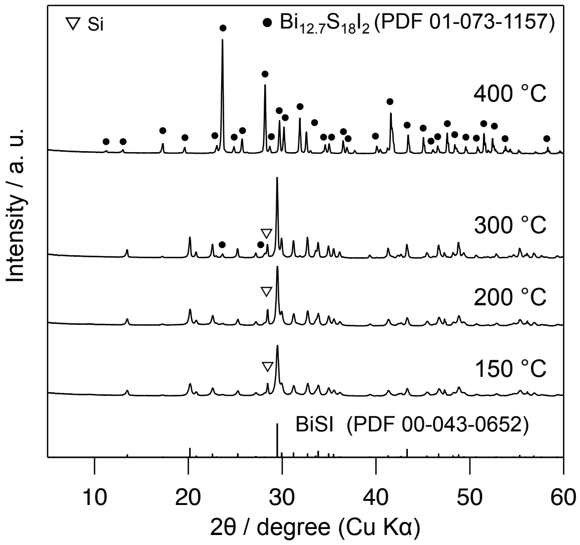

Figure S1. XRD patterns of the prepared samples at high temperature.

Table S1. Atomic % of BiOI before and after H_2S treatment as determined by EDX analysis.

	Atomic %	
	Before	After
Bi	34	34
O	33	n.d.
S	n.d.	32
I	33	33

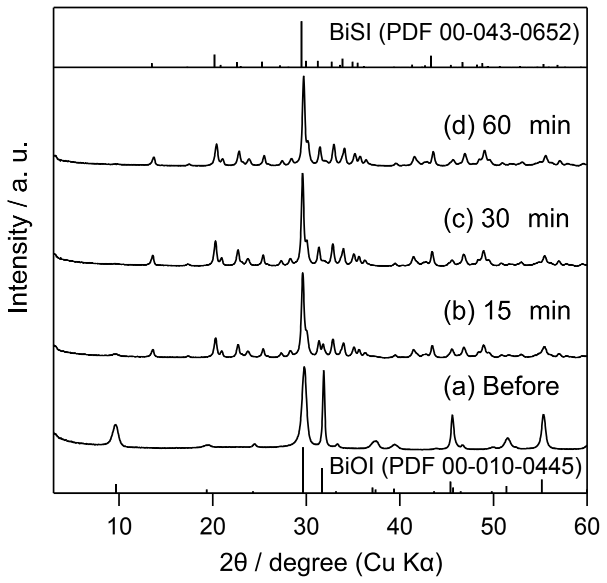

Figure S2. XRD patterns of prepared samples (a) before and after the heat treatment with 5% H_2S gas at 150 °C for (b) 15 min, (c) 30 min, and (d) 60 min.

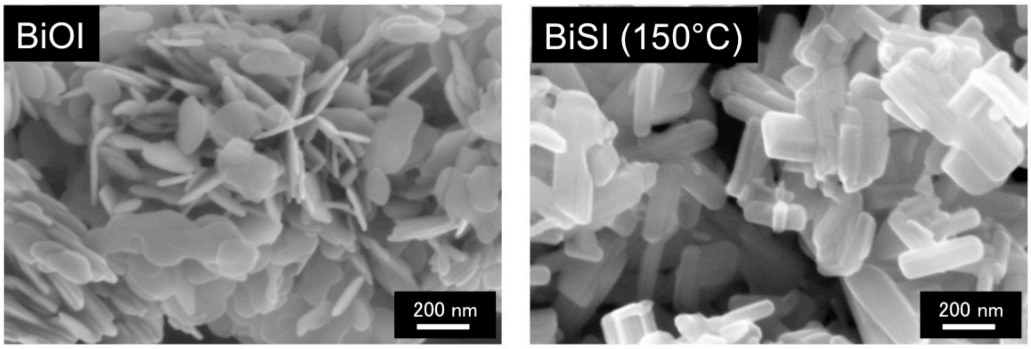

Figure S3. SEM images of prepared BiOI and BiSI (after heating under H_2S flow at 150 °C).

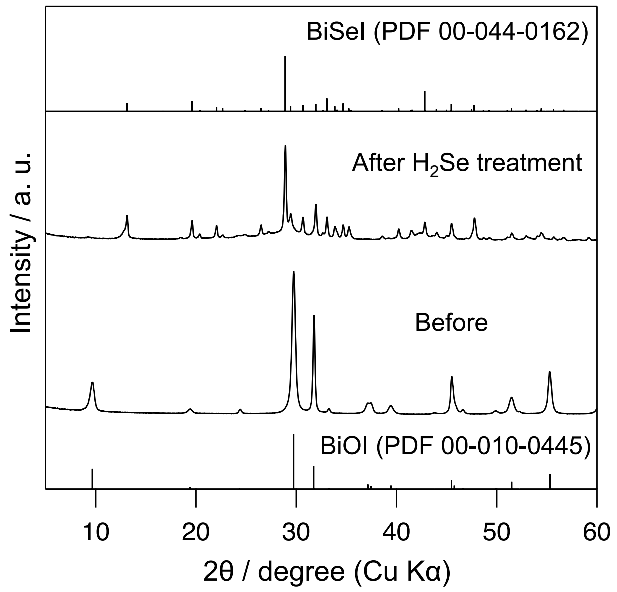

Figure S4. XRD patterns of prepared $BiSBr_{1-x}I_x$ samples by heat treatment with H_2Se gas.

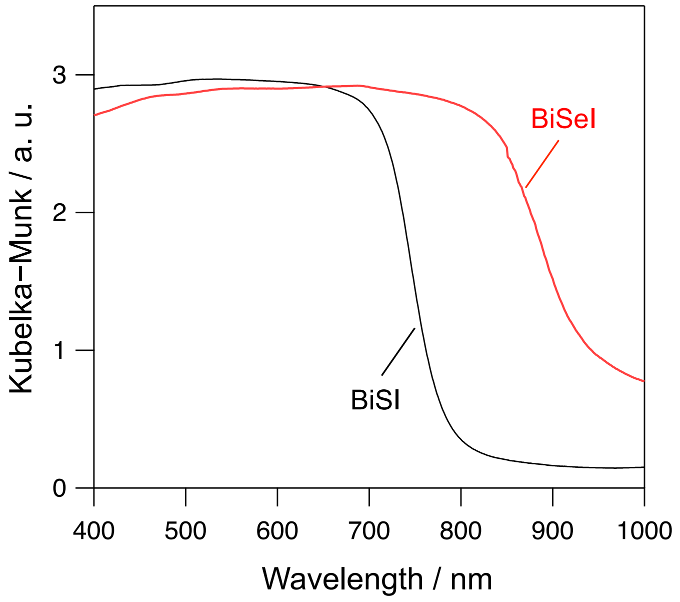

Figure S5. UV–vis diffuse reflectance spectra of BiSI and BiSeI.

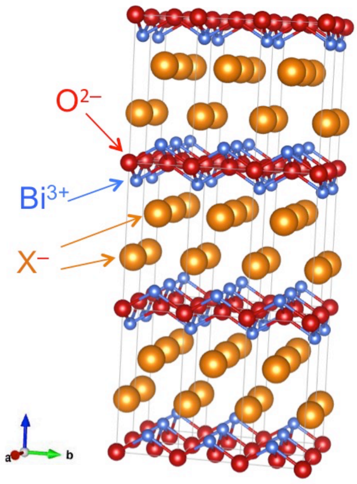

Figure S6. Crystal structure of BiOX (X = Cl, Br, I).

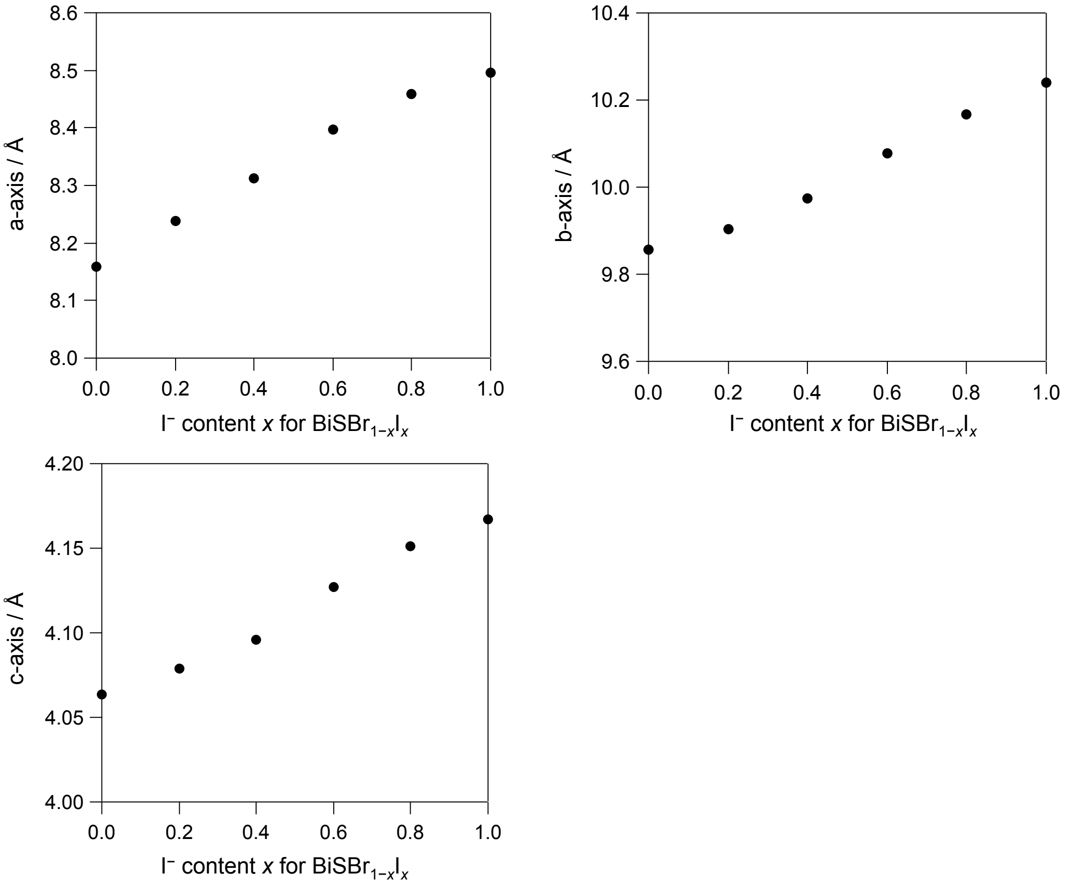

Figure S7. Lattice constants of $BiSBr_{1-x}I_x$ calculated by Le Bail analysis.

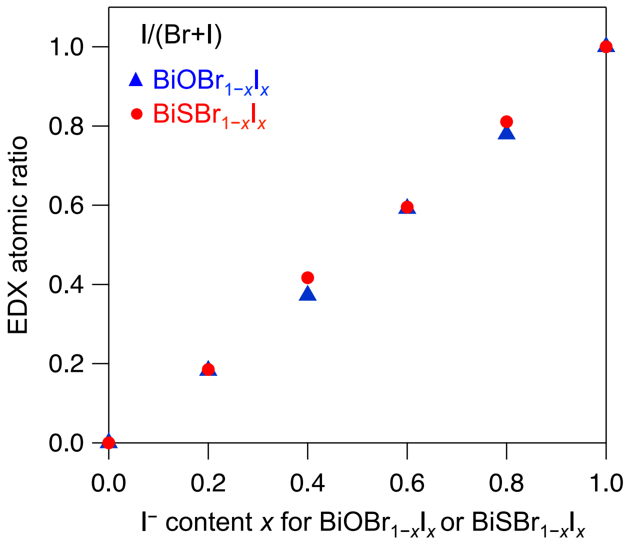

Figure S8. Atomic ratio of I to (Br+I) determined by the EDX analysis of $BiOBr_{1-x}I_x$ and $BiSBr_{1-x}I_x$.

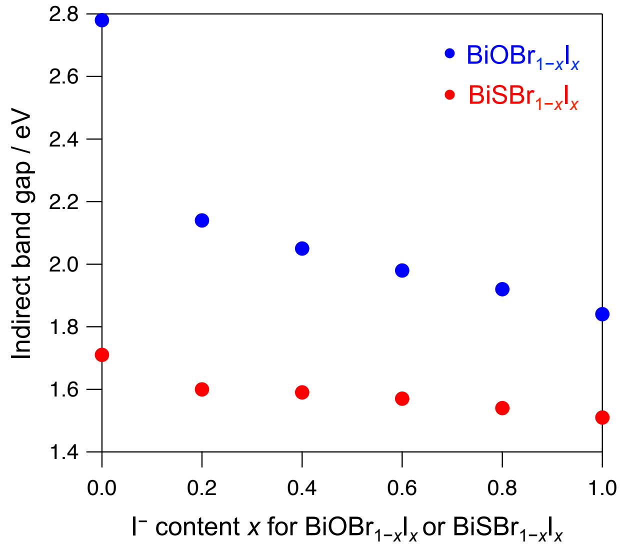

Figure S9. Indirect band gap of $BiOBr_{1-x}I_x$ and $BiSBr_{1-x}I_x$.

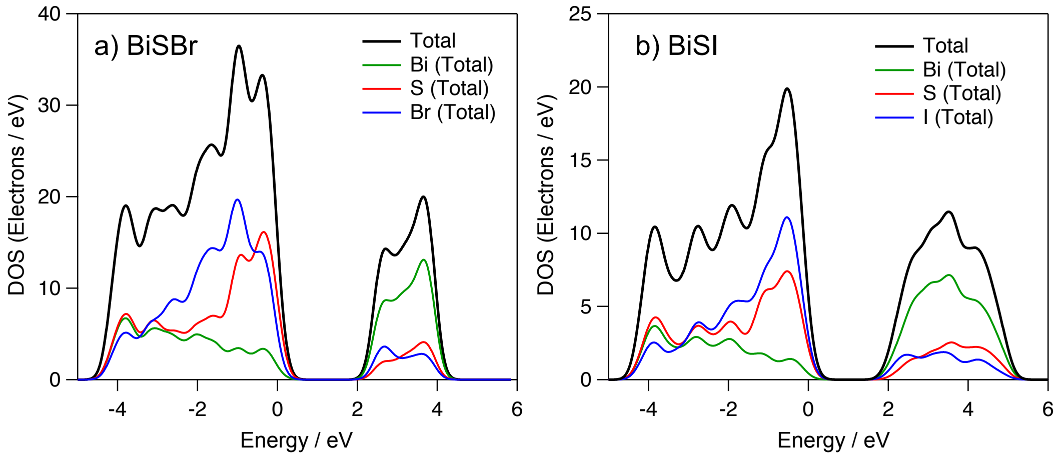

Figure S10. Density of states (DOS) of a) BiSBr and b) BiSI, and PDOS projected onto each constituent element.

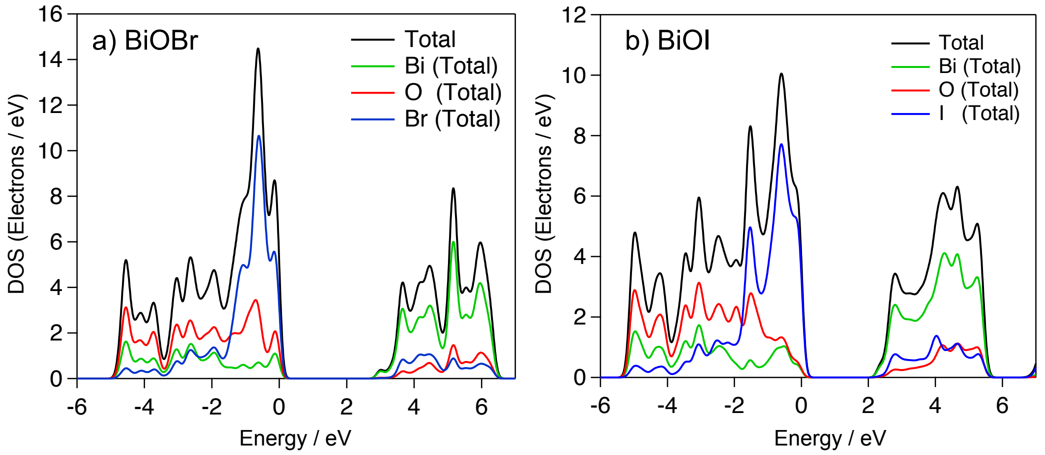

Figure S11. Density of states (DOS) of a) BiOBr and b) BiOI and, PDOS projected onto each constituent element.

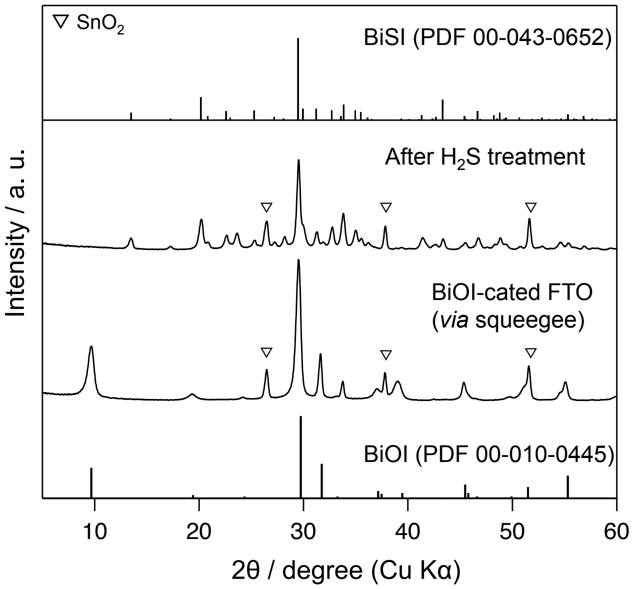

Figure S12. XRD patterns of the BiSI electrode prepared *via* the squeegee method using BiOI particles, which involves heating the BiOI-coated FTO under H_2S/Ar at 150 °C.

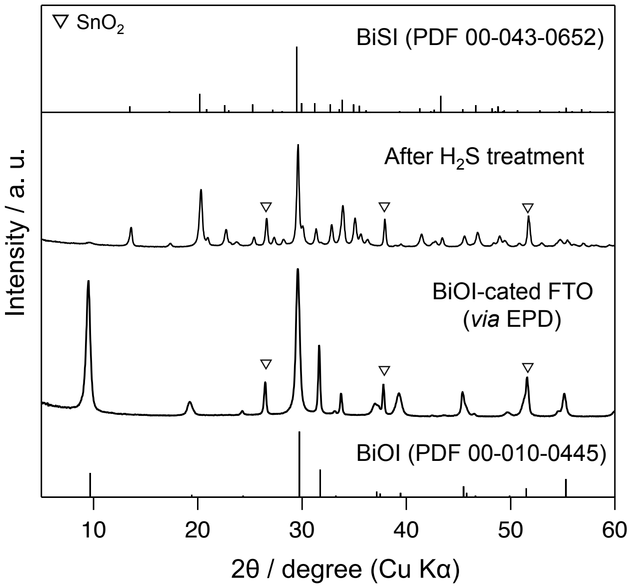

Figure S13. XRD patterns of the BiSI electrode prepared by heating the BiOI-coated FTO *via* the EPD of BiOI particles under H_2S/Ar at 150 °C.

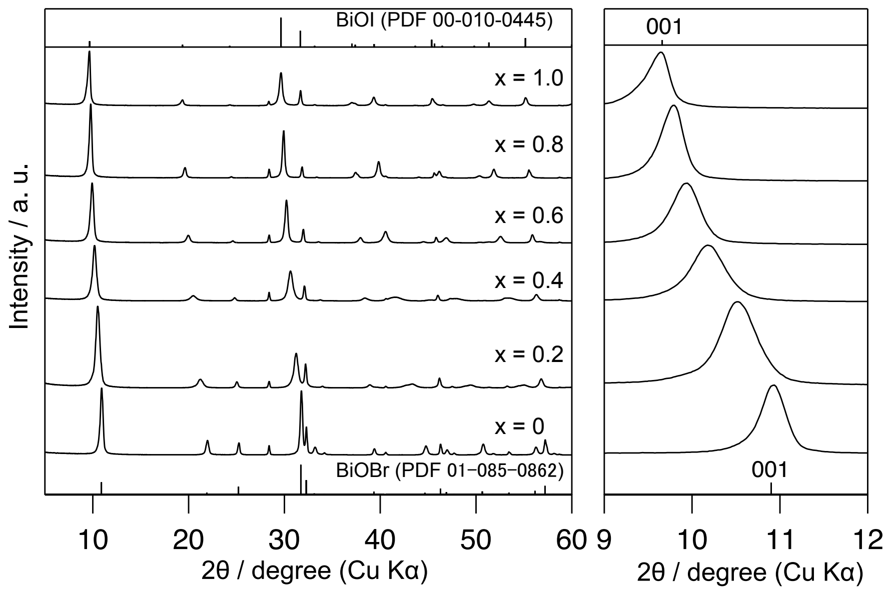

Figure S14. XRD patterns of prepared $BiOBr_{1-x}I_x$.

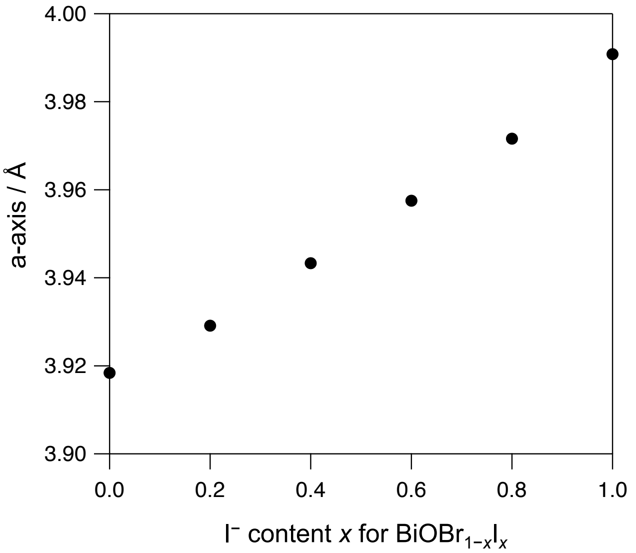

Figure S15. Lattice parameter of $BiOBr_{1-x}I_x$ calculated by Le Bail analysis.
